# Reply to: Before attributing encephalomyelitis to SARS‐CoV‐2 vaccinations exclude differentials

**DOI:** 10.1002/acn3.51468

**Published:** 2021-10-12

**Authors:** Karolina Kania, Wojciech Ambrosius, Elzbieta Tokarz Kupczyk, Wojciech Kozubski

**Affiliations:** ^1^ Department of Neurology Poznan University of Medical Sciences Poznan Poland

We appreciate interest in our case report,^1^ however, we cannot agree with the comments in Dr Finsterer's letter.^2^

Our article stated that most ADEM events might be due to infections or vaccinations. “Up to three quarters” does not mean only. Other causes of the disease are a rarity. Even letter's author cited case reports as an argument for his concerns. The presented case had no significant medical history, so other conditions have not been an issue.

Another doubt was about the test for the SARS COV‐2. We feel confused as it is stated in the text that we did RT‐PCR to detect the viral infection.

The commentary raised the question of the SARS COV‐2 vaccine as a cause of ADEM. We want to point out that our article is a case report, so it cannot be about and prove causality of any kind. There is no mention in the text that the vaccine could have caused ADEM. We observed the temporal link (nothing more) between vaccination and this disease in only one particular case. This is also an answer to the question about differential diagnosis. It is hard to exclude rare every type of encephalitis in such situation. You may always find some scarce cause within the “etcetera” group as the letter's author formulated it. We agree that the broaden differential diagnosis should be performed but rather in the larger study.

Furthermore, the MRI distinctive images of ADEM case are the counterarguments in this discussion. The MRI abnormalities mentioned by Dr Finsterer types of encephalitis have a different distribution.^3^ Oligoclonal bands were negative, which is typical in ADEM and does not impair the disease diagnosis.^4^

In response to the allegations of MRI, we presented such images after remarks from the reviewer who indicated which picture should be included.

To address the question about treatment—yes, we are sure that clinical status improved after steroids. Guidelines recommend these drugs for the ADEM treatment, which was diagnosed based on the symptoms, laboratory tests, especially MRI images.^5^ Empirical therapy with ceftriaxone and aciclovir lasted until the PCR results (negative) for common bacteria and viruses in cerebrospinal fluid were obtained.

To answer the last question, the Index IgG was 0,69.

At the end, we emphasize that the purpose of our article was to share observation of two important medical events coinciding (not cause‐and‐effect relationship) with other clinicians.

## Conflict of Interest

All authors declare that they have no conflict of interest.

## Funding Information

No funding information provided.
